# Sex Differences in the Associations of Cardiorespiratory Fitness with Executive function, Episodic Memory, and Global Cognition in Older Adults with Mild Cognitive Impairment

**DOI:** 10.1002/alz.084417

**Published:** 2025-01-09

**Authors:** Fang Yu, Dereck L. Salisbury, Keenan A Pituch, Feng Vankee Lin

**Affiliations:** ^1^ Arizona State University, Phoenix, AZ USA; ^2^ University of Minnesota, Minneapolis, MN USA; ^3^ 455 Broadway St., Redwood City, CA USA

## Abstract

**Background:**

A potential mechanism underpinning the cognitive benefits from physical activity and aerobic exercise is cardiorespiratory fitness (CRF). Greater cardiorespiratory fitness (CRF) was associated with better executive function, short‐term memory, and global cognition in older adults without cognitive impairment. Sex differences in CRF has been established in adults. However, sex differences in the associations between CRF and cognition have been limitedly studied among older adults with mild cognitive impairment (MCI).

**Method:**

This study examined sex differences in the associations of CRF with executive function, episodic memory, and global cognition in community‐dwelling older adults with amnestic MCI. Baseline data from the Aerobic exercise and Cognitive Training (ACT) Trial that tests the cognitive effects and mechanisms of 6‐month combined aerobic exercise and cognitive training were used. CRF was measured by peak oxygen consumption (VO_2peak_) in a laboratory‐based symptom‐limited peak cycle‐ergometer test. Executive function was measured with the EXAMINER that calculates 3 sub‐domain composite scores on working memory, cognitive control, and fluency, episodic memory with the Brief Visuospatial Memory Test‐Revised learning and delayed recall, and global cognition with Montreal Cognitive Assessment. Covariates included demographics, body‐mass index, comorbidities, activities of daily living, depression, and premorbid intellect. Regressions were performed.

**Result:**

On average, the participants (N = 142) were 73.8±5.8 years of age with 16.9±2.9 years of education, 87.3% White, 51.4% male, and 69.7% married. Their VO_2peak_ was 17.1±5.0 mL/kg/min. After controlling for covariates, VO_2peak_ was related to executive function (β = 0.037, SE = .015, p<.05) and episodic memory (*b* = .590, *SE* = .226, *p*<.05), but not global cognition (*b* = .074, *SE* = .055, *p* > .05). The associations of VO_2peak_ with executive function (*b* = .063, *SE* = .024, *p*<.05) and episodic memory (*b* = 1.008, *SE* = .312, *p*<.01) were significant for males only.

**Conclusion:**

Our findings show that VO_2peak_ was associated with executive function and working memory in the overall sample and males. Future studies need to examine longitudinal changes of CRF in preclinical AD and MCI to better understand of its sex‐differential impact on cognition and personalize exercise interventions on targeted cognitive domains based on sex.

